# Prediction of biological age and evaluation of genome-wide dynamic methylomic changes throughout human aging

**DOI:** 10.1093/g3journal/jkab112

**Published:** 2021-04-07

**Authors:** Mahmoud Amiri Roudbar, Seyedeh Fatemeh Mousavi, Siavash Salek Ardestani, Fernando Brito Lopes, Mehdi Momen, Daniel Gianola, Hasan Khatib

**Affiliations:** 1 Department of Animal Science, Safiabad-Dezful Agricultural and Natural Resources Research and Education Center, Agricultural Research, Education & Extension Organization (AREEO), Dezful, 333, Iran; 2 Department of Animal Science, Faculty of Agriculture Engineering, University of Kurdistan, Sanandaj, 66177-15175, Iran; 3 Department of Animal Science and Aquaculture, Dalhousie University, Truro NS B2N 5E3, Canada; 4 Department of Animal Sciences, Sao Paulo State University, Julio de Mesquita Filho (UNESP), Prof. Paulo Donato Castelane, Jaboticabal, SP 14884-900, Brazil; 5 Department of Surgical Sciences, School of Veterinary Medicine, University of Wisconsin-Madison, Madison, WI 53706, USA; 6 Department of Animal and Dairy Sciences, University of Wisconsin-Madison, Madison, WI 53706, USA

**Keywords:** aging, whole-methylome prediction, reproducing kernel Hilbert spaces, Bayesian ridge regression

## Abstract

The use of DNA methylation signatures to predict chronological age and aging rate is of interest in many fields, including disease prevention and treatment, forensics, and anti-aging medicine. Although a large number of methylation markers are significantly associated with age, most age-prediction methods use a few markers selected based on either previously published studies or datasets containing methylation information. Here, we implemented reproducing kernel Hilbert spaces (RKHS) regression and a ridge regression model in a Bayesian framework that utilized phenotypic and methylation profiles simultaneously to predict chronological age. We used over 450,000 CpG sites from the whole blood of a large cohort of 4409 human individuals with a range of 10–101 years of age. Models were fitted using adjusted and un-adjusted methylation measurements for cell heterogeneity. Un-adjusted methylation scores delivered a significantly higher prediction accuracy than adjusted methylation data, with a correlation between age and predicted age of 0.98 and a root mean square error (RMSE) of 3.54 years in un-adjusted data, and 0.90 (correlation) and 7.16 (RMSE) years in adjusted data. Reducing the number of predictors (CpG sites) through subset selection improved predictive power with a correlation of 0.98 and an RMSE of 2.98 years in the RKHS model. We found distinct global methylation patterns, with a significant increase in the proportion of methylated cytosines in CpG islands and a decreased proportion in other CpG types, including CpG shore, shelf, and open sea (*P* < 5e-06). Epigenetic drift seemed to be a widespread phenomenon as more than 97% of the age-associated methylation sites had heteroscedasticity. Apparent methylomic aging rate (AMAR) had a sex-specific pattern, with an increase in AMAR in females with age related to males.

## Introduction

Aging is a complex process with time-dependent changes in many biological functions and subject to regulation by signaling pathways and transcription factors. Reduced stability of epigenetic patterns over a lifetime in adult somatic tissue is one of the most important progressive deteriorating changes which may increase significant pathologies, including cancer, metabolic disruptions, cardiovascular malfunctions, and neurological disorders, and result in more susceptibility to death in elders (López-Otín *et al.* 2013). This instability is referred to as “aging epigenetics,” and associations between age and DNA modifications other than sequence mutations have been investigated (Fraga and Esteller 2007). Many biomarkers have been proposed for aging and age-related diseases (Baxter *et al.* 2004; Bocklandt *et al.* 2011), including telomere length shortened by cell division and oxidative stress (Sanders and Newman 2013). Another biomarker is DNA methylation, an epigenetic modification, with a robust relationship between aging and methylation changes (Heyn *et al.* 2012; Hannum *et al.* 2013; Horvath 2013; Bormann *et al.* 2016; Petkovich *et al.* 2017). DNA methylation changes can impact the aging rate by altering the expression of age-related genes via the silencing of DNA repair mechanisms or silencing anti-inflammatory genes.

Predicting quantitative traits with regression models for dense molecular markers—such as high-throughput genotyping data, sequencing information, and methylation profiling microarrays—is important in animal and plant breeding schemes (de los Campos *et al.* 2013; Hu *et al.* 2015) and human genetics (de los Campos *et al.* 2010a; Vazquez *et al.* 2016; Amiri Roudbar *et al.* 2020). When fitting high-density molecular markers in prediction models, the number of predictors dramatically exceeds the number of observations. Several variable selection methods or shrinkage estimation procedures have been proposed for nonparametric or parametric regression models (Gianola *et al.* 2006; de los Campos *et al.* 2013). Most complex traits can be predicted with reasonable accuracy using the infinitesimal model, which assumes a vast number of loci contributing to the trait, each with an infinitesimal effect (Fisher 1919; Buckler *et al.* 2009; Yang *et al.* 2010). Many DNA methylation marks show significant associations with age (Heyn *et al.* 2012; Hannum *et al.* 2013), suggesting that the infinitesimal models may also be useful for predicting chronological age. Gianola *et al.* (2006) presented a semiparametric procedure, named reproducing kernel Hilbert spaces (RKHS) regression, to predict quantitative traits that makes use of phenotypic and molecular information simultaneously. RKHS provides a flexible framework for integrating high-dimensional multilayer omic-data to predict survival after diagnosis of breast cancer (Vazquez *et al.* 2016). Bayesian methods are powerful alternative approaches for predicting complex traits using variable selection and shrinkage of estimates. Various Bayesian parametric linear regressions that differ in their priors have been proposed for genome-enabled prediction [see Gianola (2013) and de los Campos *et al.* (2013) for discussions of Bayesian regressions]. Bayesian ridge regression (BRR) performs a homogenous shrinkage across markers. Statistically, BRR on markers is equivalent to genomic best linear unbiased prediction (GBLUP), but with variance parameters estimated Bayesianly (VanRaden 2008; Gianola 2013).

The use of DNA methylation signatures to predict the aging rate and chronological age is of interest to many fields, including disease prevention and treatment, forensics, and evaluation of anti-aging drugs. In recent years, many methods have been proposed to predict an individual’s chronological age (Bocklandt *et al.* 2011; Hannum *et al.* 2013; Bekaert *et al.* 2015; Naue *et al.* 2017; Vidaki *et al.* 2017). However, most of these studies used a small number of markers selected based on previously published studies or used methylation information retrieved from 27 or 450 K microarrays for marker selection. Selected markers, ranging from 3 to 513, have been implicated in age prediction using multivariate linear regression models (Hannum *et al.* 2013; Hong *et al.* 2017; Levine *et al.* 2018) or nonparametric methods such as machine learning algorithms (Xu *et al.* 2015; Vidaki *et al.* 2017).

Semiparametric and Bayesian approaches for age prediction have not been evaluated so far. Here, we evaluated RKHS and BRR models for whole methylome prediction of age in humans using a large cohort of 4409 individuals composed of four publicly available DNA methylation datasets representing a range of 10–101 years of age. Furthermore, marker subset selection was carried out using epigenome-wide association study (EWAS) results to detect age-associated methylation sites. Breusch-Pagan test (BPtest) results were used to retrieve age-associated methylation sites without heteroscedasticity. Finally, to better understand how the methylome changes with age, we evaluated genome-wide methylomic profiling with this large cohort of individuals, including the effect of sex of the individual.

## Materials and methods

**Figure 1:**
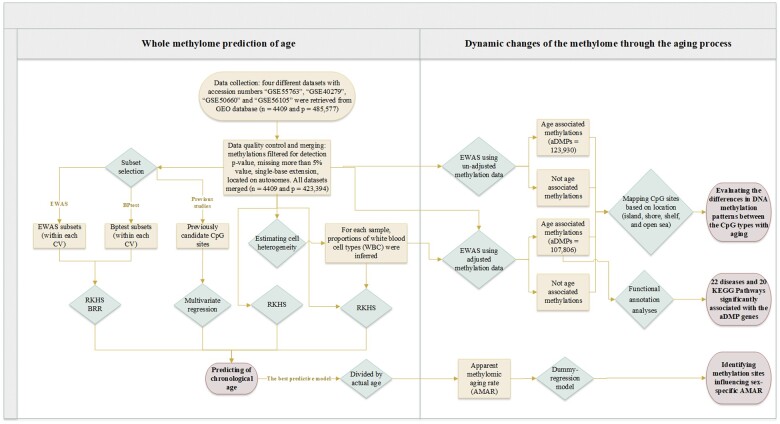
Flow chart for prediction analyses (left) and detection of dynamic changes on methylation level (right).

The steps of the prediction analysis and detection of the dynamic methylomic changes are presented in Figure 1.

### Whole methylome prediction of age

#### Datasets

Methylation data were from four different datasets. All data are available in Gene Expression Omnibus (GEO) with accession numbers “GSE55763,” “GSE40279,” “GSE50660,” and “GSE56105” (Supplementary File S1, Supplementary Table S1 and Supplementary Figure S1). DNA extraction, preparation procedures, and DNA methylation profiling were previously described for each dataset separately (Hannum *et al.* 2013; McRae *et al.* 2014; Tsaprouni *et al.* 2014; Lehne *et al.* 2015). Briefly, to assess the methylation levels of over 485,000 CpG sites per sample, DNA extracted from whole blood was treated with bisulfite and then hybridized to the Illumina Infinium 450 k Human Methylation Beadchip. In each dataset, individual methylation values with detection *P*-value >0.01 were set as missing values. Probes unsuccessfully measured in 5% of samples, with SNPs at CpG or single-base extension (SBE) sites and located on the X and Y chromosomes were excluded from the analyses. Cross-reactive probes were also removed. Missing values were imputed using the R “impute” package with 10 nearest neighbors averaging (Troyanskaya *et al.* 2001). After data preprocessing, all datasets were merged, and probes that were common in all datasets were kept. In the end, 423,394 methylation sites from 4409 individuals remained for training and validation of the models. For each CpG site, the beta value was used to indicate the percentage of methylation. The values ranged from 0 to 1 for entirely unmethylated and fully methylated, respectively. After merging the datasets, we used the Z-score conversion to standardize the methylation levels.

#### Estimating cell heterogeneity for each sample

The methylation profiles were measured in DNA extracted from whole blood with heterogeneous cell proportions, which can act as a potential confounder when investigating DNA methylation differences over a wide age range (Jaffe and Irizarry 2014). We used the Houseman *et al.* (2012) linear regression calibration to estimate the relative proportion of pure cell types. For each sample, the proportions of white blood cell (WBC) types, including granulocytes, monocytes, B cells, CD4+ T cells, CD8+ T cells, and natural killer cells, were inferred using 473 CpGs which had previously shown cell-type-specific methylation patterns (Reinius *et al.* 2012). This was performed using the ChAMP R package (Tian *et al.* 2017). The estimated cell-type distributions were used to adjust methylation beta values using the regression model.

As shown in previous studies, cell proportions adjustment can reduce association signals; therefore, it is essential to consider cell composition variability in epigenetic studies based on whole blood and other heterogeneous tissue sources (Liu *et al.* 2013; Jaffe and Irizarry 2014). We tested the association of each cell type proportion with age using a linear model with sex and dataset considered as fixed effects. Cell type proportion was not associated with aging (*P* > 0.05, Supplementary File S1 Supplementary Tables S2–S7). Therefore, the final model for EWAS employed the un-adjusted methylation data.

#### Statistical models for predicting chronological aging

Parametric and semiparametric approaches were used to predict chronological aging. A statistical and computational challenge was that the number of methylation sites exceeds the number of data points. Therefore, shrinkage estimation procedures were applied (Meuwissen *et al.* 2001; Gianola *et al.* 2006). Predictive ability using the entire methylation data set and subsets of it was evaluated with RKHS regression (Gianola and van Kaam 2008; Morota and Gianola 2014) and Ridge Regression (Hoerl and Kennard 1970) (BRR) in a Bayesian framework.

##### Reproducing kernel Hilbert space

RKHS regression is a powerful semiparametric approach to cope with the issues of dimensionality and complexity raised by a massive number of predictors (De los Campos *et al.* 2010b). We built a covariance structure among methylation values and treated age as a continuous response using the following kernel regression model:

y=Xb+Kα+e,

where b is a vector of fixed effects including an intercept and sex and dataset effects with associated incidence matrix X, K is an n×n positive definitive kernel matrix indexed by adjusted or un-adjusted methylation levels, α is the vector of RKHS regression coefficients estimated as the solution of $\;\hat{\alpha }=\underset{\alpha }{\min}\left\{{\left(y-K\alpha \right)}^{'}\left(y-K\alpha \right)+\lambda \alpha^{'}K\alpha \right\}$, where λ is a regularization parameter and e is the vector of independently distributed residual effects. Here α and e were assumed as random vectors with distributions $\alpha ∼N(0,\;K^{-1}\sigma_{m}^{2})$ and$e∼N(0,\;{I\sigma }_{e}^{2})$, where $\sigma_{m}^{2}$ and $\sigma_{e}^{2}$ are the methylomic and the residual variances, respectively, and I is an identity matrix. RKHS was fitted using the Gaussian kernel function:

$$K\left(m_{i},\;m_{i^{'}}\right)=\exp\left[-h\;\left(\frac{\sum_{j=1}^{p}{\left(m_{ij}-m_{i^{'}j}\right)}^{2}}{p}\right)\right],$$

where $m_{i},\;m_{i^{'}}$ are vectors of methylation measurements in the i and $i^{'}$ individuals (*i *=* *1, 2, …, *n*) for p methylation sites (j=1, …,p), h is a bandwidth parameter chosen with cross-validation (CV), based on their ability to predict a testing set. There was no significant difference in accuracy between bandwidths less than 1. The best bandwidth was 0.03, which was used for further analyses. RKHS was fitted using the Bayesian likelihood under Gaussian assumptions:

$$p(y\theta )=\;\prod_{i=1}^{n}N(y_{i}|\sum_{k=1}^{l}x_{ik}b_{k}+\sum_{j=1}^{p}k_{ij}\alpha_{i},\sigma_{e}^{2})$$

where θ is the vector of unknown parameters, including the intercept, regression coefficients ($b_{k}$), residual variance ($\sigma_{e}^{2}$), and $k_{ij}$ is the appropriate element of K.

##### Bayesian ridge regression

We also used a whole-genome Bayesian Ridge regression approach (Meuwissen *et al.* 2001). A Gaussian prior distribution (BRR) was assigned to methylation effects to control the shrinkage of estimates. Age was considered as a continuous response with the following model equation in matrix notation:

y=Xb+Mu+e,

where $M=\{m_{ij}\}$ is a matrix of un-adjusted beta values in the *i*th individual at *j*th methylation site (*j *=* *1, …, p), and u is a vector of the corresponding methylation site effects. The conditional distribution of the data was:

$$p(y\theta )=\;\prod_{i=1}^{n}N(y_{i}|\sum_{k=1}^{l}x_{ik}b_{k}+\sum_{j=1}^{p}m_{ij}u_{j},\sigma_{e}^{2}),$$

where θ is the vector of all unknown parameters with the following prior density:

$$p(\theta )=p(\sigma_{e}^{2})\prod_{k=1}^{l}p(b_{k})\prod_{j=1}^{p}p(u_{j}).$$

The fixed effects were assigned a flat prior, the residual variance was assigned a scaled-inverse X^2^ density with degrees of freedom df_e_ and scale parameter S_e_ using the default treatment of variances implemented in the BGLR R package (Pérez and de los Campos 2014). The methylation effects, $u_{j}$, were assigned a Gaussian prior with variance $\sigma_{u}^{2}$.

#### Subset selection of CpG sites

In the full models used, there were 4409 samples with more than 420,000 methylation sites as predictor variables. Although variable selection methods or shrinkage estimation procedures can handle a larger number of predictors than the sample size, pre-subset selecting of methylation sites may increase the performance of the predictive model (Amiri Roudbar *et al.* 2020).

##### EWAS subset selection

A previous association study involving methylation sites and chronological age revealed about 15% significant methylation sites (Hannum *et al.* 2013). Here, we applied an EWAS subset selection approach to evaluate the impact of the number of predictors on prediction [for model description, see “EWAS Analyses to identify age-associated differentially methylated positions (aDMPs)” section]. For each CV sample, we conducted an EWAS using training data only to find aDMPs until each CV has aDMP subsets. These subsets were used to contrast predictions with those attained with random subsets of methylation probes, *i.e.*, fitting the age-associated methylation positions (aDMPs) versus randomly selected methylation sites.

##### BPtest subset selection

Each aDMP, detected using only the training set, was tested for heteroscedasticity using the BPtest model (Breusch and Pagan 1979). For this test, only methylation information from the training set was used. Methylation trends due to age, sex, and dataset were removed by fitting a linear model for each aDMP, and the residuals were retrieved. Next, the BPtest was performed by fitting a linear regression model to these residuals. aDMPs without heteroscedastic disturbances were selected based on Benjamini-Hochberg’s false discovery rate (FDR) less than 0.05 (Benjamini and Hochberg 1995). The influence of this selection approach on prediction accuracy was evaluated against randomly selected probes in each CV.

##### Subset selection of CpG Sites based on previous studies

We used results from three studies to fit prediction models based on their candidate CpG sites (Horvath 2013; Horvath *et al.* 2018; Levine *et al.* 2018). The “optimal” model from these studies selected 353 (Horvath multi-tissue method) (Horvath 2013), 391 (Horvath skin method) (Horvath *et al.* 2018), and 513 (Levine method) (Levine *et al.* 2018) methylation markers that were highly predictive of age. We missed some of these methylation sites due to quality control. The number of missed CpG sites and their designations are shown in Supplementary File S1, Supplementary Table S8. Measurements of these candidate CpG sites in each method were used to predict chronological age using the multiple regression model:

$$y_{i}=\sum_{k=1}^{l}x_{ik}b_{k}+\sum_{s=1}^{h}m_{is}u_{s}+e_{i},$$

where $u_{s}$ is the methylation effect of the sth site from the subset selected based on the three methods listed above.

#### Prediction accuracy

We used two measures of predictive accuracy. The first one was Pearson’s correlation between predicted age and chronological age. The second was the root mean square error (RMSE). RMSE measures closeness between chronological and predicted ages and correlation measures association.

To assess the prediction accuracy of chronological age, fivefold CVs were performed. In each fold, 20% of each dataset was missed randomly. The whole fivefold CV was repeated four times, producing a total of 20 samples. For each CV, 5000 posterior samples were used to compute the posterior means of the parameters. Averages of correlation and RMSE of age from the 20 CVs were used to compare the models. All models were fitted using the “BGLR” R package (Pérez and de los Campos 2014). We used the coda package diagnostic tests of convergence in the Bayesian analyses (Plummer *et al.* 2006). To evaluate the effects of dataset and age on prediction accuracy, we also fitted a simple linear regression with only these potential confounders as model covariates.

### Dynamic changes of the methylome through the aging process

#### EWAS analyses to identify age-associated differentially methylated positions (aDMPs)

We fitted a linear regression of chronological age on each CpG site separately. As methylation levels may be affected by cell populations' heterogeneity, the effects of differential cell count on aDMPs were examined using adjusted and un-adjusted beta values. Sex and dataset were treated as fixed effects in the model. The genetic background of each individual was not available, except for “GSE40279”. Due to this limitation, the genetic background was not used as a potential confounder in the linear model. Adjusted and un-adjusted beta values were fitted one-at-a-time using the following linear model:

y=Xb+ma+e

where m is the vector of the adjusted/un-adjusted beta values at a CpG site, and a is the fixed methylation effects. A stringent Bonferroni-adjusted threshold was used to correct for multiple testing (0.05/423,394 = 1.18e-7).

#### Mapping CpG sites and dynamics through the aging process

CpG islands (CGIs) are, on average, 1000 base pairs (bp) long and can be distinguished from other genomic regions by being GC-rich, CpG-rich, and mostly unmethylated. They are frequently associated with more than 70% of the promoter region of genes (Deaton and Bird 2011). CpG shores are sequences up to 2 kb distant from CGIs. Most tissue-specific differential methylation in normal tissues occurs more frequently in CpG shores than in CpG islands (Irizarry *et al.* 2009). CpG regions were further classified by including CpG shelves as sequences 2–4 kb distant from CGIs, and CpG open sea as more than 4 kb distant from CGIs (Bibikova *et al.* 2011). We used the IlluminaHumanMethylation450kanno.ilmn12.hg19 package from Bioconductor (Gentleman *et al.* 2004) to group CpG probes located on the same island, shore, shelf, or open sea. Methylation markers in each CpG type were classified into two groups: (1) age-associated and (2) not age-associated methylation sites. To evaluate differences in DNA methylation patterns between the groups due to aging, we fitted a linear regression of the average individual methylation levels in each group at different genome locations. We fitted a linear model as follow:

$${\overset{-}{\mathrm{Beta}}}_{i}=\sum_{k=1}^{l}x_{ik}b_{k}+\vartheta_{i},$$

where ${\overset{-}{\mathrm{Beta}}}_{i}$ is the average of individual methylation levels in age-associated or not age-associated methylation sites at a CpG type (island, shore, shelf, or open sea), and $\vartheta_{i}$ is an independent normal model residual with mean zero and variance$\;\sigma_{\vartheta }^{2}$.

#### Functional annotation analyses

Functional annotation for the selected methylation sites was performed using pathway and disease association analyses. For each methylation site, the R package minfi was used to access annotation for each position (Aryee *et al.* 2014). Then, genes associated with aDMPs were characterized employing BioMart web services through the R package biomaRrt (Durinck *et al.* 2009). Gene lists retrieved from each group were uploaded to the Database of Annotation, Visualization, and Integrated Discovery (DAVID; david.abcc.ncifcrf.gov) v6.8 ^42^ to link them to associated diseases and pathway using genetic association database (GAD) source (Becker *et al.* 2004) and KEGG pathway maps (Kanehisa and Goto 2000), respectively.

#### Methylation sites influencing sex-specific apparent methylomic aging rate (AMAR)

AMAR for each individual was computed using results of the best predictive model, RKHS with EWAS subset selected CpG sites, but without a sex variable. AMAR was calculated as the individual’s predicted age for the best model, divided by actual age. It is known that sex can affect the aging rate derived from a methylome profile, with a faster aging rate in males than in females (Hannum *et al.* 2013). Methylomic aging rate in males was 4% faster than in females. Here, we included sex as a dummy explanatory variable to account for a sex effect on methylation levels across chronological ages. To test differences between male and female methylome aging rates, first, we fitted the three linear models as follows:

Model 1: ${\mathrm{AMAR}}_{i}=\beta_{0}+\beta_{1}A_{i}+\tau_{i}$

Model 2: ${\mathrm{AMAR}}_{i}=\beta_{0}+\beta_{1}A_{i}+\beta_{2}S_{i}+\tau_{i}$

Model 3:$\;{\mathrm{AMAR}}_{i}=\beta_{0}+\beta_{1}A_{i}+\beta_{2}S_{i}+\beta_{3}S_{i}A_{i}+\tau_{i}$

where ${\mathrm{AMAR}}_{i}$ is an estimated response variable for the *i*th individual; $A_{i}$ is chronological age for the *i*th individual; $S_{i}$ is the dummy explanatory variable for sex; $\beta_{0}$, $\beta_{1}$, $\beta_{2}$, and $\beta_{3}$ are the intercept, age, male effect, and interactions between sex and age (difference between male and female slope over aging), respectively; and $\tau_{i}$ is an independent normal model residual with mean zero and variance $\sigma_{\tau }^{2}$. Models were compared using the Akaike information criterion (AIC) (Akaike 1998). Model 1 states that there is no difference between males and females in AMAR over aging, but Models 2 and 3 state that there are sex differences and interaction between sex and age, respectively.

Second, differences in methylome changes over a wide age range in males and females were tested at each aDMP. Slopes were compared using the model:

$$A_{id}=\beta_{0}+D_{d}+\beta_{4}M_{i}+\beta_{2}S_{i}+\beta_{5}S_{i}M_{i}+\varepsilon_{id}$$

where $A_{id}$ is age for the *i*th individual in the *d*th dataset, $D_{d}$ is the fixed effect of the *d*th dataset, $M_{i}$ is methylation level for the *i*th individual, and $S_{i}M_{i}$ is a joint-effect covariate. $\beta_{4}$ and $\beta_{5}$ are the methylation effect and interactions between sex and methylation levels, respectively, and $\varepsilon_{ij}$ is an independent normal residual with mean zero and variance $\sigma_{\varepsilon }^{2}$. Sex-specific aDMP associations were corrected for multiple testing using the inclusion threshold based on the Benjamini-Hochberg at a desired FDR less than 0.05 (Benjamini and Hochberg 1995).

### Data availability

The Illumina 450 K methylation array datasets analyzed are publicly available in GEO with accession numbers “GSE55763,” “GSE40279,” “GSE50660,” and “GSE56105.” Supplementary material is available at figshare: https://doi.org/10.25387/g3.14356925.

## Results

### The whole methylome prediction model

Prediction accuracy metrics using the linear model with only the fixed effects, including age and dataset, were 11.244 and 0.733 for RMSE and correlation between real and predicted age, respectively. These results suggest that part of correlation accuracy may reflect dataset and gender effects (confounders) due to differences in age distribution among the datasets and between genders. Methylation age could be sensitive to factors, such as sex (Hannum *et al.* 2013). Accordingly, we examined epigenome-wide prediction using adjusted and un-adjusted beta values according to the proportion of WBCs, sex, and dataset. Then, different subsets of methylation markers, including EWAS, BPtest, and randomly selected markers were investigated.

#### Effect of adjustment for estimated WBC heterogeneity on age prediction accuracy

**Figure 2:**
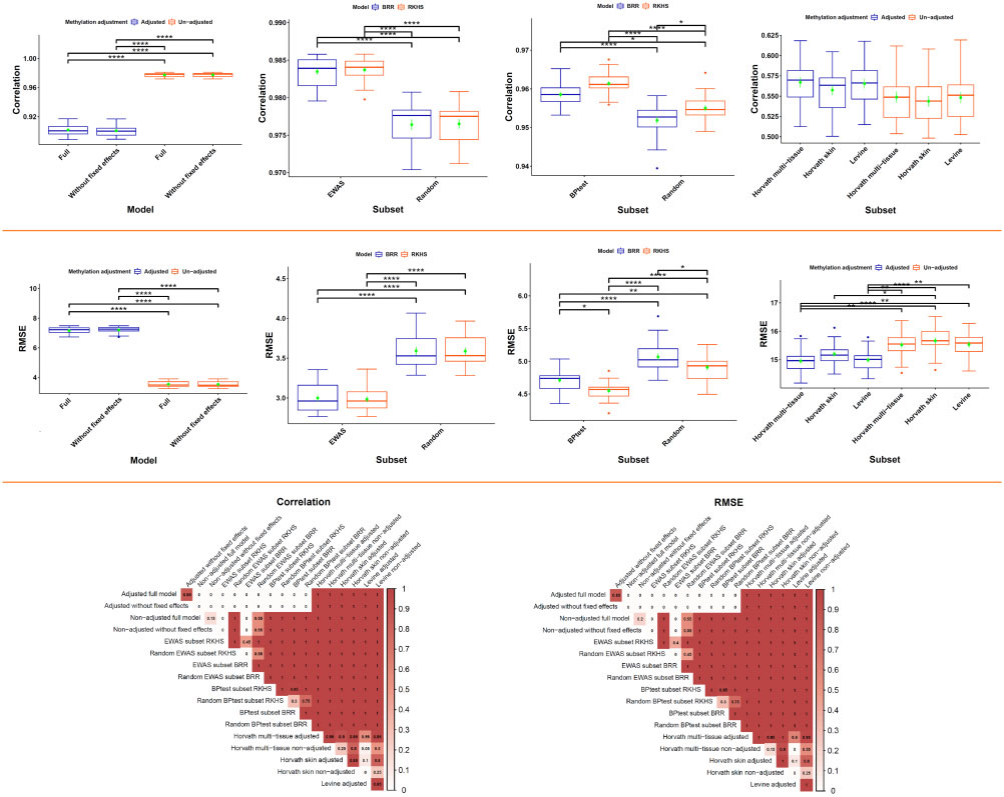
Boxplot of average cross-validation of predictive accuracy (A) correlation and RMSE (B) between chronological and predicted age for different methods using different subsets of methylation measurements with mean and standard deviation (green) and significance levels; (C) proportion of times that model in row had correlation (right) and RMSE (left) more and less, respectively, than model in color.

The use of un-adjusted methylation produced higher prediction accuracy with RKHS than adjusted methylation data (Figure 2). Therefore, we used un-adjusted methylation data for subsequent analyses.

#### Predictive ability of different methylation subsets

Model complexity, prediction accuracy, and goodness of fit for each type of subset selection are given in Figure 2. EWAS subset selection seemed to improve prediction accuracy, as the lowest RMSE and the highest correlation were achieved in this subset. There were no significant differences between semiparametric and parametric approaches using EWAS selection. The correlation of predicted age between the two approaches was very high (Supplementary Figure S2).

We tested each aDMP for heteroscedasticity using a BPtest and excluded aDMPs with nonnormally distributed and serially independent residuals. Markers whose residual variance showed a change with age are illustrated in Supplementary File S1, Supplementary Figure S3. Of 123,930 aDMPs, 121,023 markers showed heteroscedasticity (FDR < 0.05), and over 97% of these markers showed an increase for independent residuals with age (see Supplementary File S1, Supplementary Table S9 for details). For BPtest subset prediction, after excluding aDMPs with heteroscedasticity, prediction accuracy from CV analyses was relatively high when using about 2000 CpG sites (number changed in each CV). Average correlations between absolute and predicted age from 20 CVs were 0.96 (with 4.55 years RMSE) and 0.96 (with 4.71 years RMSE) for RKHS and BRR, respectively. These estimates were about 0.7% higher than those obtained when the same number of randomly selected methylation sites (random BPtest subset) was used. In this subset selection method, prediction accuracies were 0.2% (significantly) higher for RKHS than for BRR but did not differ in RMSE. Selecting subsets of methylation markers with EWAS produced a gain in prediction accuracy.

We applied these methods considering cell heterogeneity using adjusted and un-adjusted methylation measurements. The correlation between chronological and predicted age using adjusted (un-adjusted) measures were 0.57 (0.55), 0.56 (0.54), and 0.57 (0.55) for Horvath multi-tissue Horvath skin, and Levine methods, respectively (Figure 2A). RMSE was 14.9, 15.2, and 15.0 years, respectively, when adjusted measurements were used (Figure 2B). These accuracies were significantly lower than those obtained using RKHS and BRR (*P*  < 0.05).

### Dynamic changes of the methylome with aging

#### Age-associated methylation signatures

**Figure 3:**
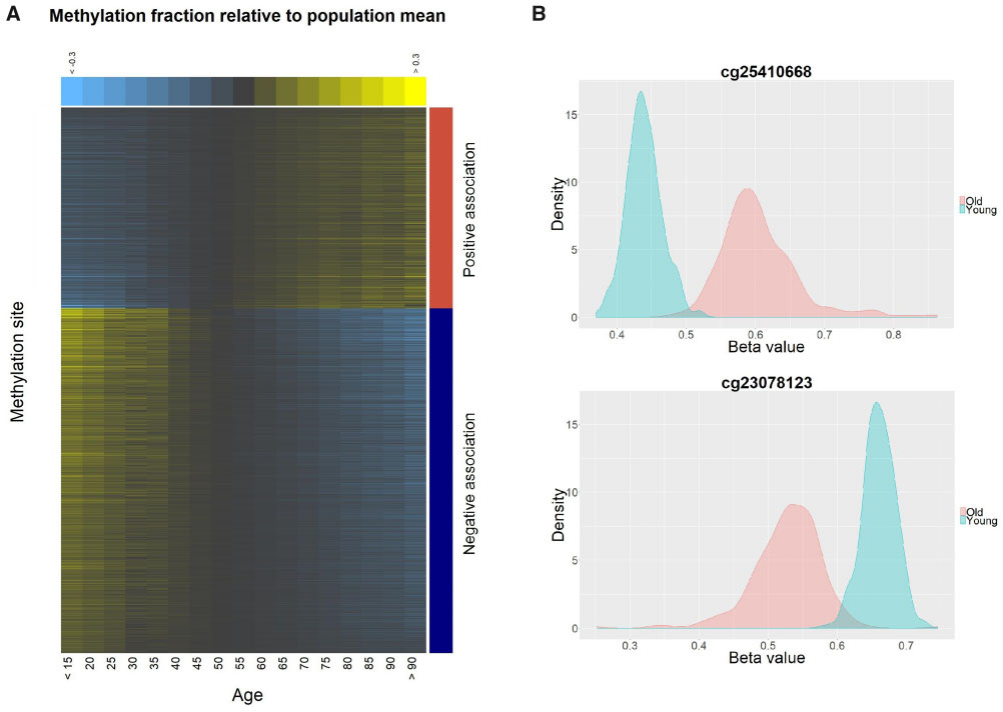
Un-adjusted methylation profiles by age. (A) Heatmap of the top 1000 age-associated methylation markers. CpG sites are sorted by the magnitude of regression coefficients. Individuals are grouped into intervals of 5 years and ordered from the youngest to the oldest group. (B) Density plots of beta values by age group for positively (top) and negatively (bottom) age-associated methylation markers in the 10% youngest (iris blue) and 10% oldest (salmon) individuals.

Results indicated that the number of probes, significantly associated with age, was reduced from 123,930 probes in un-adjusted data to 107,806 probes in adjusted EWAS (Figure 3). This vast number of age-associated markers points to the complexity of aging, and these sites can be used for future studies.

#### Location-specific dynamic changes of the methylome with aging

**Figure 4:**
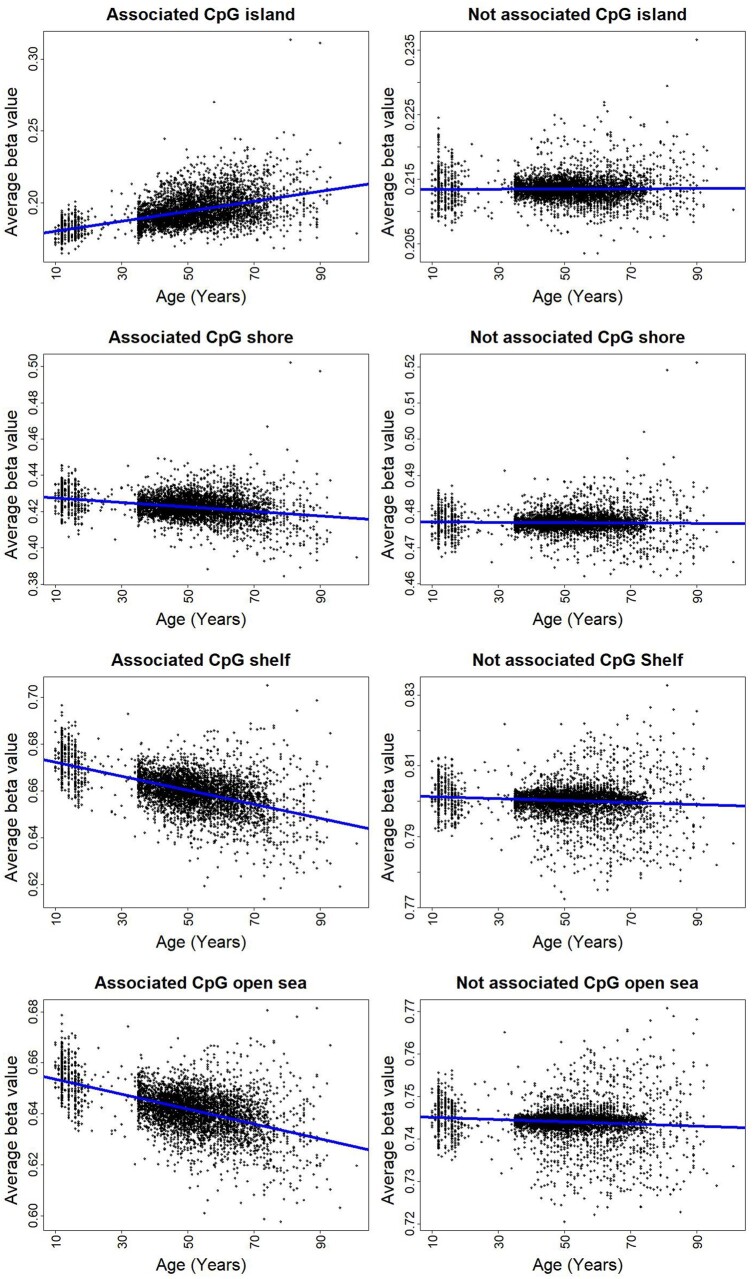
Plots of regressions of average of individual methylation levels at different locations in the genome on age. Plots illustrate association of average beta values and age calculated from the four CpG types, including CpG island (A), shore (B), shelf (C), and open sea (D). Methylation markers in each CpG type classified into two categories: age-associated and not-associated methylation sites. In age-associated sites, the global methylation level increased and decreased with age in CpG island and off CpG island, respectively (*P* < 5e-06). In not-associated methylation sites, CpG shelf and open sea were significantly demethylated with increasing age (*P* < 0.01).

EWAS revealed that about 30–33% of age-associated methylation sites were found in adjusted and unadjusted data and CGIs had a larger proportion of positive associations (83%, see Supplementary File S1, Supplementary Table S10). The proportion of positive associations drastically decreased in CpG shore, shelf, and open sea areas, both with adjusted and unadjusted beta values. To investigate methylation patterns in different types of CpG sites, we calculated the average of age-associated and not-associated methylation beta values for each CGI type separately and evaluated changes of average methylation with aging (Figure 4). Significant differences in global methylation levels between males and females were observed in all CpG types, with an increase in the proportion of methylation at CGI and decreased proportion at CpG shore, shelf, and open sea in females compared to males (Supplementary File S1, Supplementary Tables S11–S14).

#### Functional annotation of aDMPs

We investigated 10,596 genes with aDMPs for their various disease associations. A total of 6740 genes were found to be associated with a disease based on GAD annotation. We identified 22 diseases to be significantly associated with the aDMP genes (FDR < 0.05). The most significant related disorder was tobacco use disorder, with 2177 related genes (Supplementary File S1, Supplementary Table S15). The GAD analysis revealed other age-related diseases associated with aDMP genes such as type 2 diabetes (Rönn *et al.* 2008), erythrocyte count (Detraglia *et al.* 1974), body mass index and related disorders (*e.g.*, waist circumference and body weight) (Hochberg *et al.* 1995; Poehlman *et al.* 1995), cholesterol (Kreisberg and Kasim 1987), triglycerides (Tucker and Turcotte 2003), cardiovascular diseases (*e.g.*, heart rate, heart failure, stroke, coronary artery disease, blood pressure, and arteries) (Taddei 2009; Breitling *et al.* 2012) and Parkinson disease (Levy 2007) (Supplementary File S1, Supplementary Table S15). These results also indicated that age-related DNA methylation modifications in 312 genes were related to alcoholism disorders.

A total of 3328 genes were found to be associated with KEGG Pathways that have been previously related to aging (FDR < 0.05) (Supplementary File S1, Supplementary Table S16). For instance, the most significant KEGG pathway was the focal adhesion. The aging process in humans is associated with reduced flexibility of joints and tissue elasticity caused mainly by alterations in focal adhesion formation on the surface of cells (Arnesen and Lawson 2006). The second significant KEGG pathway was the regulation of the actin cytoskeleton, which plays a fundamental role in cellular pathways (Mooren *et al.* 2012). A total of 142 genes were significantly associated with this KEGG pathway. It has been shown that aging can reduce cytoskeletal integrity and that overexpression of the heat-shock transcription factor, HSF-1, plays a role in the preservation of the cytoskeleton (Baird *et al.* 2014).

#### Methylome aging rate and sex-specific methylomic aging rate (AMAR)

**Figure 5:**
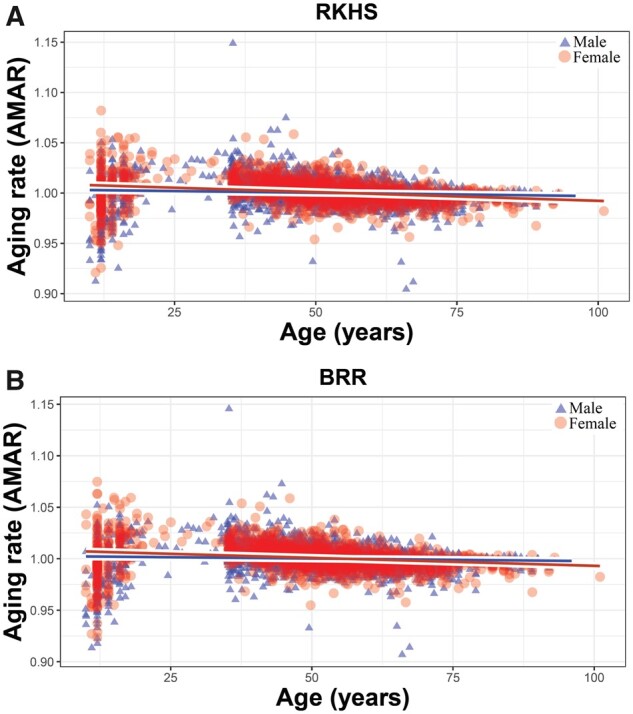
Regression of AMAR on age in males (blue) and females (red). Models were (A) reproducing kernel Hilbert spaces (RKHS) regression and (B) BRR; both models showed sex-specific AMAR patterns. In both approaches, females had significantly slower AMAR than males (*P* < 0.01; see Supplementary File S1, Supplementary Table S17).

The AIC showed that sex significantly affected the methylomic aging rate in BRR and RKHS (Supplementary File S1, Supplementary Table S17). As shown in Figure 5, the AMAR was significantly reduced in females than males by age (*P <* 0.01). This result confirms a previous study, which showed a faster aging rate in males (Hannum *et al.* 2013). A total of 1549 suggestive sites were identified, which may influence AMAR producing sex-specific methylation patterns (Supplementary File S2).

## Discussion

Recent studies have applied elastic net regression models and artificial neural networks to predict chronological age using methylation clock sites (Hannum *et al.* 2013; Horvath 2013; Petkovich *et al.* 2017; Stubbs *et al.* 2017; Vidaki *et al.* 2017). These methods automatically select a small subset of methylation markers (maximum a few hundred) to fit a model that is highly predictive of chronological age. In contrast, our study found that the number of methylation sites related to age is over 100,000. It appears more sensible to use an age prediction model where all methylation sites are fitted simultaneously. Consequently, we compared two parametric and semiparametric methods. BRR was used as a parametric method to fit a massive number of methylation sites by assuming a Gaussian prior distribution of the effect of each methylation site (de los Campos *et al.* 2013). We chose RKHS as a semiparametric method, with a Gaussian kernel to predict chronological age (Morota and Gianola 2014).

Although BRR and RKHS use a different type of shrinkage of estimates to handle the problem of a large number of the predictors relative to sample size, there was no significant difference between these models. These results are in agreement with a previous study that found no difference in whole-genome prediction accuracies between parametric and semiparametric approaches in maize (Riedelsheimer *et al.* 2012). The slight difference between BRR and RKHS suggests that there is a little or no interaction effect between methylated CpG sites, that RKHS regression is not able to capture it. Using EWAS, we found that most methylation sites had no association with age, and subset selection increased the performance of predictive, with a smaller number of predictors. To further evaluate whole methylome prediction using RKHS and BRR, three previously presented procedures—Horvath multi-tissue, Horvath skin method, and Levine method—were also evaluated. Fitting all methylation sites simultaneously yielded better predictions of age. The accuracy was slightly higher than reported in previous studies that used the elastic net approach (Hannum *et al.* 2013; Horvath 2013). This increase in accuracy could be due to the large sample size used in our study, fitting all aDMPs simultaneously, or the different methodologies used in these studies.

As whole blood is a heterogeneous ensemble of white cells, with each type having a different epigenetic profile, adjustment for WBC heterogeneity in EWA studies is strongly recommended when this type of tissue is used. Heterogeneous cell populations in the blood may act as a potential confounder when cell distribution differs over target traits. For instance, adjustment for WBC proportions reduced the strength of association signals in rheumatoid arthritis disease (Liu *et al.* 2013). Although EWASs have illustrated the importance of adjusting for changes in cell-type composition (Liu *et al.* 2013; Jaffe and Irizarry 2014), the impact on whole methylation prediction has not been studied in age prediction. However, adjusting methylation profiles for cellular heterogeneity had a detrimental effect on the classification ability of rheumatoid arthritis cases from controls (Amiri Roudbar *et al.* 2020). In concordance, we found a decrease in prediction accuracy from using the adjusted methylation data related to un-adjusted methylation data.

It has been suggested that methylomic aging rates vary among different individuals (Hannum *et al.* 2013) and that DNA methylation changes accumulate over the lifetime. Hannum *et al.* (2013) reported 27,800 markers with heteroscedasticity, and 99.8% showed an increase in variation with age. Our results indicate that epigenetic drift is a more extensive phenomenon than generally believed.

Global DNA methylation level decreases with aging in mammalian tissues (Wilson and Jones 1983). The methylation of a CGI located in the promoter region of the *estrogen receptor* (*ER*) gene was associated with age in normal colorectal mucosa (Issa *et al.* 1994). Furthermore, a vast proportion of age-related methylation sites in normal breast tissue was located in CGIs, and a close relationship between age-related DNA methylation changes and epigenetic alterations was present in breast tumors (Johnson *et al.* 2014). In a study on a cancer-free population, no significant differences in global DNA methylation were found between different middle age groups (Zhang *et al.* 2011). Our results indicate that methylation sites located in CGIs tend to gain methylation with increasing age, whereas aDMPs located in other CpG types, including shore, shelf, and open sea areas, tend to lose methylation with aging. Interestingly, the average methylation level of aDMPs located in CpG islands was significantly higher in older individuals. With increasing distance from CpG islands, the average methylation levels for aDMPs significantly decreased with aging. These results agree with previous studies that found age-related methylation sites to be methylated and unmethylated preferentially at CpG islands (Christensen *et al.* 2009) and CpG shores (Horvath 2013), respectively. The average methylation level for nonassociated methylation sites changed slightly and remained constant over the age range. These results enabled us to identify distinct age methylation patterns in CpG types according to distance from CGIs. This global methylation pattern revealed that age-related changes in DNA methylation are not distributed randomly on the genome. Understanding the mechanisms that produce these location-specific methylation patterns during the human lifespan warrants further research.

Recently, a significant difference in average methylation levels between males and females was observed in individuals ranging from 41 to 55 years of age (Tsang *et al.* 2016). A significantly lower methylation level was found for males than for females. In contrast, in a study of human peripheral blood from individuals ranging from 45 to 75 years, females had lower methylation levels (Zhang *et al.* 2011). With these contradictory reports, the global DNA methylation variation between sexes remains unclear, although it is accepted that females undergo a slower aging rate, as they have a slower methylomic aging rate (Hannum *et al.* 2013) and a later age-related decline (Gur and Gur 2002). We found that the average methylation at CGI increased with age, but the average methylation at CpG shore, shelf, and open sea decreased in elders. The differences in methylation patterns between males and females may result from a slower methylomic aging rate in females. These results suggest that regions located near to and far from CGIs tend to be methylated and demethylated, respectively, over aging in a sex-specific manner and that the global sex-specific methylation level may change across the genome.

One limitation in this study was the use of cohorts with different age and sex distributions for age prediction. Consequently, a portion of the correlation between actual and predicted age might be due to confounders, given that the model with only sex and dataset achieved a higher correlation (0.733) than the prediction models based on three previous studies with gender and dataset (0.557–0.570 for adjusted data). This suggests that using a small number of CpG sites to fit an age prediction model would not be an appropriate choice when including confounders that influence prediction. However, our results showed that using a whole-genome methylome prediction approach may overcome this limitation and could be a better choice for predicting chronological age with these datasets.

Using methylation profiles to predict chronological age has potential applications in many fields, including disease prevention and treatment, forensics, and anti-aging medicine. A predicted biological age can also be used to estimate aging rates, which help explain epigenetic drift (Hannum *et al.* 2013). Most of the age-prediction methods use a small number of methylation sites, although many of these CpG sites may not be methylated. Therefore, using whole-genome methylome prediction models in this study may lead to a more realistic aging rate prediction.

Age-related diseases such as metabolic syndrome, obesity, type 2 diabetes mellitus, and cardiovascular diseases are increasing due to the growing aging population observed worldwide. Several mechanisms play essential roles in the development of age-related diseases, including epigenetic processes (Franceschi *et al.* 2018). To assess the functional relationship between epigenetics and age-related diseases, genes associated with aDMPs were analyzed by disease association analysis. Smoking can affect DNA methylation, and some of the influenced genes are involved in the risk of age‐related conditions such as cardiovascular diseases (Breitling *et al.* 2012; Dogan *et al.* 2014). Our results indicate that the accumulation of epigenetic changes with age was comparable to smoking‐associated DNA methylation changes, which implies that smoking can likely change the dynamic of the DNA methylation pattern similar to the aging process. Results of our pathway analysis indicate that age-associated epigenetic landscape alterations may contribute to disease susceptibility through biological pathways.

Genetic and environmental factors can either hasten or delay the aging process. Several aging phenotypes have been associated with genetic variation within the *FOXO3A* gene (Willcox *et al.* 2008), and some of these genetic variants are associated with a slowing or delay in age-related disease (Kenyon 2010). Lifestyle choices such as smoking and physical fitness are among the environmental factors that can influence the aging process (Blair *et al.* 1989). Sex can also affect human longevity, as mortality rates are higher for males, and females tend to live longer than males in most countries (Austad 2006). These observations have led to the search for molecular markers of aging rate, and a faster methylomic aging rate in males than in females has been reported (Hannum *et al.* 2013). Although females can live longer than males, there is a limited number of studies attempting to understand the mechanisms that drive changes in the aging rate in a sex-specific manner. Methylomic assessment of sex-specific age-related methylation sites allows us to understand differences in methylomic aging between sexes. We identified over 1500 putative methylation sites that could affect variation in methylomic aging rate. This large number of sex-specific methylated aDMPs highlights the complexity of the methylomic aging rate.

## Conclusions

In this study, we found that choosing age-associated methylation sites according to EWAS for pre-selection of predictors helps improve prediction ability. Further research on pre-selection methods may help to find even more accurate prediction models. The use of cell-type corrections needs to be carefully considered in chronological age predictions. Interestingly, We found that the methylation patterns of age-associated sites differ with their genome position. There is an increase in methylation at CGIs, whereas CpG shore, shelf, and open sea show decreased methylation levels with age. We found that methylation sites can display a sex-specific methylation rate. These sites may help explain the increased methylomic aging rate for males. However, understanding the molecular mechanisms underlying sex-specific aging processes warrants further research.
